# Genetic and Epigenetic Interplay Define Disease Onset and Severity in Repeat Diseases

**DOI:** 10.3389/fnagi.2022.750629

**Published:** 2022-05-03

**Authors:** Lise Barbé, Steve Finkbeiner

**Affiliations:** ^1^Center for Systems and Therapeutics, Gladstone Institutes, San Francisco, CA, United States; ^2^Department of Neurology, University of California, San Francisco, San Francisco, CA, United States; ^3^Department of Physiology, University of California, San Francisco, San Francisco, CA, United States

**Keywords:** epigenetics, genetics, nucleotide repeat disease, trinucleotide repeat disease, methylation, repeat instability, chromatinization, CTCF

## Abstract

Repeat diseases, such as fragile X syndrome, myotonic dystrophy, Friedreich ataxia, Huntington disease, spinocerebellar ataxias, and some forms of amyotrophic lateral sclerosis, are caused by repetitive DNA sequences that are expanded in affected individuals. The age at which an individual begins to experience symptoms, and the severity of disease, are partially determined by the size of the repeat. However, the epigenetic state of the area in and around the repeat also plays an important role in determining the age of disease onset and the rate of disease progression. Many repeat diseases share a common epigenetic pattern of increased methylation at CpG islands near the repeat region. CpG islands are CG-rich sequences that are tightly regulated by methylation and are often found at gene enhancer or insulator elements in the genome. Methylation of CpG islands can inhibit binding of the transcriptional regulator CTCF, resulting in a closed chromatin state and gene down regulation. The downregulation of these genes leads to some disease-specific symptoms. Additionally, a genetic and epigenetic interplay is suggested by an effect of methylation on repeat instability, a hallmark of large repeat expansions that leads to increasing disease severity in successive generations. In this review, we will discuss the common epigenetic patterns shared across repeat diseases, how the genetics and epigenetics interact, and how this could be involved in disease manifestation. We also discuss the currently available stem cell and mouse models, which frequently do not recapitulate epigenetic patterns observed in human disease, and propose alternative strategies to study the role of epigenetics in repeat diseases.

## Introduction

Nucleotide repeat expansions that extend the normal length of disease genes cause neuronal and neuromuscular diseases (Schmidt and Pearson, 2016). Most repeat diseases are caused by an expansion of trinucleotide repeats, typically a “CXG” expansion. Repeat diseases can originate from expansions in exons, introns, promoter regions, or the 5′ or 3′-UTR of genes (Ellerby, 2019). All classical repeat diseases involve neuronal dysfunction in patients, often accompanied by neuromuscular symptoms (Ellerby, 2019). Most repeat diseases are autosomal dominant, with an increase in disease severity and decrease in age of disease onset over successive generations (Ashizawa et al., 1992a,b). In addition, beyond classical repeat diseases, expanded tandem repeats are also thought to contribute to heritability in some cases of polygenic diseases, such as autism spectrum disorder (ASD) (Hannan, 2010).

Nucleotide repeats are found throughout the genome, but only certain repeat expansions cause disease (Sun et al., 2018). The mechanisms determining which repeat expansions lead to cellular dysfunction and disease remain unknown. In addition, the strength of the correlation of repeat expansion length to disease severity or age of onset varies and can be low. However, repeat length is not the only determining factor for the expression of symptoms in these diseases. Patients with similar repeat lengths can have very different ages of onset, progression and symptoms (Cobo et al., 1993; Barcelo et al., 1994; Clark et al., 1998; Langbehn et al., 2004; Wexler et al., 2004; De Antonio et al., 2016; Keum et al., 2016). Therefore, other mechanisms are thought to play a role in determining the effect repeat expansions have on cellular function and disease phenotype.

Methylation near a disease-implicated repeat region was first reported in Fragile X Syndrome, a trinucleotide repeat disease involving a CGG expansion in the 5′-UTR of the fragile X mental retardation I (*FMR1*) gene (Verkerk et al., 1991). Methylation at the promoter region of *FMR1*, upstream of the CGG repeat, silences the gene and causes neuronal dysfunction in patients (Oberle et al., 1991; Pieretti et al., 1991; Hansen et al., 1992; Sutcliffe et al., 1992; O’Donnell et al., 2002). Several other repeat diseases also show epigenetic changes in methylation, chromatin conformation and gene downregulation at the repeat regions.

In this review, we focus on six repeat diseases: myotonic dystrophy type I (DM1), fragile X syndrome (FXS), Friedreich ataxia (FRDA), amyotrophic lateral sclerosis (ALS), Huntington disease (HD), and spinocerebellar ataxias (SCA). Despite the different patterns of pathology, disease progression and onset, DNA methylation near repeat regions, CTCF binding and chromatin conformation changes are common in all these diseases (Table 1), and are linked to disease severity. Additionally, we discuss the interplay between genetics and epigenetics and their role in repeat instability and the evidence that expanded tandem repeats and increased methylation and chromatinization also contribute to ASD. Finally, we review the disease models that are currently available to study repeat diseases and the emerging importance of using transdifferentiated cells to study diseases that involve epigenetic mechanisms and aging.

**TABLE 1:** A comparison of repeat diseases with different genetic characteristics shows a common epigenetic signature.

	DM1	FXS	FRDA	ALS (C9ORF72)	HD	SCA
gene	DMPK	FMR1	FXN	C9ORF72	HTT	SCA genes
repeat	CTG	CGG	GAA	GGGGCC	CAG	CAG
disease repeat length	50–5,000	50–200 premutation, >200 full mutation	100–1,200	30–1,600	35–120	33–80
repeat region	3′-UTR	5′-UTR	intron 1	intron 1	exon 1	coding exons
methylation increase	YES (Steinbach et al., 1998; Lopez Castel et al., 2011; Naumann et al., 2014; Yanovsky-Dagan et al., 2015; Franck et al., 2021)	YES (Oberle et al., 1991; Hansen et al., 1992; Sutcliffe et al., 1992; Chiurazzi et al., 1998; Lanni et al., 2013)	YES (Al-Mahdawi et al., 2008, 2013; vans-Galea et al., 2012; Quesada et al., 2015; Russ et al., 2015; Wang et al., 2017)	YES (Jones and Laird, 1999; Laffita-Mesa et al., 2012; Liu et al., 2014; Gijselinck et al., 2016; Hamzeiy et al., 2018)	YES (Lanni et al., 2013)	YES (Libby et al., 2008; De Souza et al., 2016)
loss of CTCF binding	YES (Thornton et al., 1997; Naumann et al., 2014)	YES (Sun et al., 2018)	YES (Al-Mahdawi et al., 2008; Russ et al., 2015)	?	YES (Xi et al., 2013)	YES (Tremblay and Jiang, 2019)
heterochromatinization	YES (Eriksson et al., 2001; Yanovsky-Dagan et al., 2015; Buckley et al., 2016)	YES (Coffee et al., 1999; Gheldof et al., 2006; Biacsi et al., 2008; Kumari and Usdin, 2010)	YES (Gheldof et al., 2006; Rai et al., 2008)	YES (Gijselinck et al., 2012)	?	?
loss of TAD boundary	YES (Sun et al., 2018)	YES (Sun et al., 2018)	YES (Sun et al., 2018)	YES (Sun et al., 2018)	YES (Sun et al., 2018)	YES (SCA1) (Sun et al., 2018)
gene downregulation	YES (Fu et al., 1993; Hofmann-Radvanyi and Junien, 1993; Sabouri et al., 1993; Shaw et al., 1993; Jansen et al., 1996; Benders et al., 1997; Berul et al., 1999; Eriksson et al., 2000, 2001; Inukai et al., 2000; Klesert et al., 2000; Mounsey et al., 2000; Sarkar et al., 2000; Depardon et al., 2001; Coffee et al., 2002)	YES (Tabolacci et al., 2005; Biacsi et al., 2008; Colak et al., 2014)	YES (Gheldof et al., 2006; Al-Mahdawi et al., 2008; De Biase et al., 2009; vans-Galea et al., 2012)	YES (DeJesus-Hernandez et al., 2011; Gijselinck et al., 2012; Waite et al., 2014; Thomson and Leavitt, 2018)	NO	NO

## Genetics

Nucleotide repeat sequences called microsatellites are short stretches of bases that are repeated 5–50 times, and thousands of microsatellites are found across the genome (Richard et al., 2008). Microsatellites are useful for parental and cell line fingerprinting and cancer diagnosis (Gulcher, 2012). In repeat diseases, disease-specific microsatellites that occur in coding or non-coding regions are expanded past their typical length, with expansion exceeding a certain threshold resulting in disease. Repeat diseases show anticipation, a worsening of the disease and earlier disease onset in successive generations. Anticipation is explained by the inheritance of expanded repeats and an increase in repeat length in the offspring (Ridley et al., 1988; Ashizawa et al., 1992a,b; Shelbourne et al., 1992; Sutherland and Richards, 1992; Tsilfidis et al., 1992; Sano et al., 1994; Komure et al., 1995; La Spada, 1997; Nolin et al., 2003; Pearson, 2003; Hsiung et al., 2012). Furthermore, some individuals inherit pre-mutations, which are intermediate repeat lengths that do not induce disease symptoms in the individuals but do increase the chance of developing disease-causing expanded repeats in their offspring (Goldberg et al., 1993; Myers et al., 1993; Yamagata et al., 1994; Cossee et al., 1997; Stevanin et al., 1998; Tassone et al., 2000; Martorell et al., 2001; Aubry et al., 2008; Bourgeois et al., 2009; Nordin et al., 2015; Xi et al., 2015). Interruptions in the repetitive sequence have been found in a small subpopulation of patients for most repeat diseases. Interruptions tend to stabilize the repeat, and individuals with repeat interruptions show less dramatic disease phenotypes and later disease onset compared to patients with similar, but pure, repeat lengths (Matsuyama et al., 1999; Zuhlke et al., 2002; Stolle et al., 2008; Musova et al., 2009; Yrigollen et al., 2012; Hu et al., 2017; Ciosi et al., 2019; Genetic Modifiers of Huntington’s Disease (GeM-Hd) Consortium, 2019; Wright et al., 2019). Changes in repeat length within one individual are caused by slippage of DNA repair proteins on DNA loops, generated as a result of long repetitive sequences (Usdin et al., 2015; Schmidt and Pearson, 2016). This constant expansion and contraction of the repeat is called repeat instability, and results in different repeat lengths across affected tissues and even on a cell-to-cell basis within one tissue. The protective effect of repeat interruptions is likely due to increased repeat stability, since the interruptions prevent slippage of the DNA repair proteins (Gacy et al., 1995; Pearson et al., 1998a,b, 2005; Sobczak and Krzyzosiak, 2005; Kovtun and McMurray, 2008). Repeat size and instability is greater in disease-affected organs and tissues (Anvret et al., 1993; Ashizawa et al., 1993; Thornton et al., 1994; Chong et al., 1995; Dobkin et al., 1996; Takano et al., 1996; Watanabe et al., 2000; Kennedy et al., 2003; De Biase et al., 2007; Nordin et al., 2015; Mouro Pinto et al., 2020). Mismatch DNA repair proteins are thought to be the key regulators of repeat instability, and genetic variants in these genes are associated with a change in age of disease onset (Genetic Modifiers of Huntington’s Disease (GeM-Hd) Consortium, 2015, 2019; Bettencourt et al., 2016; Morales et al., 2016; Lee et al., 2017; Moss et al., 2017; Flower et al., 2019).

DM1 is caused by an expanded CTG repeat that exceeds 50 repeats in the 3′-UTR of the DM1 protein kinase (*DMPK*) gene (Aslanidis et al., 1992; Brook et al., 1992). Repeat size is somewhat correlated with disease severity (Yum et al., 2017). However, repeat lengths in individuals with the most severe form, congenital DM1, range from 500 to 3,000, which overlaps significantly with repeat length in individuals with the adult-onset form of the disease, which can range from 60 to 1,200 repeats (Cobo et al., 1993; Barcelo et al., 1994; Clark et al., 1998; De Antonio et al., 2016). This makes accurate prediction of disease phenotype and disease course based on repeat length impossible, and indicates that factors other than the repeat size play a role in disease phenotype and age of disease onset.

FXS is caused by a CGG repeat that exceeds 50 repeats in the 5′-UTR of the *FRM1* gene (Verkerk et al., 1991). Disease phenotypes are strictly divided into two categories by a cut-off length of 200 repeats. Individuals with an expansion below 200 repeats develop fragile X-associated tremor/ataxia (FXTAS), with onset of muscular phenotypes followed by cognitive impairment in late adulthood. Expansion beyond 200 repeats is called a “full mutation,” and results in disease onset during childhood that includes excessive motor impairment and intellectual disability. Methylation at the FMR1 promoter region is specifically associated with full mutation alleles, and is the striking difference between FXTAS and FXS, highlighting the role epigenetics can play in the severity of disease manifestation (Jin and Warren, 2000).

FRDA is caused by a GAA repeat expansion beyond 100 repeats in the first intron of the frataxin (*FXN*) gene (Campuzano et al., 1996). In FRDA, repeat size is only minimally correlated with disease onset and disease phenotype. In fact, the shorter of the two alleles is more significantly correlated with age of disease onset than the expanded allele, and explains 25–50% of the variance in age of disease onset with a given expanded repeat (Filla et al., 1996; La Pean et al., 2008). Approximately 25% of patients have an unpredictable genotype-phenotype correlation. This is thought to be influenced by genetic background, interrupted GAA repeats, repeat instability and potentially other molecular signatures such as epigenetics (Montermini et al., 1997; Sharma et al., 2004; De Biase et al., 2007, 2009; Stolle et al., 2008).

One of the familial forms of ALS is caused by a hexanucleotide repeat GGGGCC (G4C2) repeat expansion in the first intron of the chromosome 9 open reading frame 72 (*C9ORF72*) gene (DeJesus-Hernandez et al., 2011). Interestingly, this same G4C2 expansion is also the single greatest genetic cause of frontotemporal dementia and the same families can get either disease or a mixture of symptoms of both (DeJesus-Hernandez et al., 2011). A phenotype-genotype correlation between repeat expansion size and ALS disease severity has not been established.

HD is caused by a CAG expansion of over 35 repeats in exon 1 of the *HTT* gene (MacDonald et al., 1993). Although correlation exists between repeat size and age of disease onset and death (Langbehn et al., 2004, 2010; Keum et al., 2016), only 60% of the age of onset can be predicted by CAG length itself (Wexler et al., 2004), and correlation between repeat length and disease progression is poor in common alleles (between 40 and 50 repeats) (Keum et al., 2016). Additionally, genetic variants in mismatch repair genes can change disease onset, potentially by influencing repeat instability (Genetic Modifiers of Huntington’s Disease (GeM-Hd) Consortium, 2015, 2019).

SCA is a collection of diseases, some of which are caused by expanded CAG repeats in the coding region of various genes. For ataxias SCA1, SCA2, SCA3, SCA6, SCA7, SCA17 and dentatorubral-pallidoluysian atrophy, expansion beyond 39, 33, 45, 20, 34, 41, and 35 repeats in the *ATXN1*, *ATXN2*, *ATXN3*, *CACNA1A*, *ATXN7*, *TBP*, and *ATN1* genes, respectively, will cause disease. Pathologic repeat sizes are relatively small for most CAG repeat spinocerebellar ataxia disorders, and research on the correlation between molecular repeat characteristics and disease phenotype is limited. For SCA1, the number of CAG repeats is correlated with disease severity, and additional molecular characteristics, such as repeat interruption, are thought to play more of a role (Goldfarb et al., 1996; Matsuyama et al., 1999; Menon et al., 2013). Therefore, prediction of disease onset and severity is most accurate when considering the stretch of uninterrupted CAG repeats (Matsuyama et al., 1999). SCA17 patients typically have interrupted repeats, and the total length of the repeat is counted as a combination of the number of CAG and CAA repeats at the repeat locus. Atypical cases with uninterrupted repeats show increased disease severity, onset and anticipation of the disease in successive generations (Zuhlke et al., 2003).

ASD is predominantly a polygenic disorder, although some cases have been linked to mutations in the *FMR1* gene, which is expanded in FXS (McCary and Roberts, 2013), and others to repeat expansions in *DMPK*, *FXN*, and *HTT*, which cause DM1, FRDA, and HD, respectively (Piras et al., 2020; Trost et al., 2020). In addition, an increase in expanded tandem repeats in genes related to neuronal development and function was recently described in children with ASD compared to controls (Trost et al., 2020; Mitra et al., 2021). Lastly, mutations in genes encoding proteins important in chromatin regulation and epigenetic machinery are also observed in higher prevalence in ASD patients (De Rubeis et al., 2014). Taken together, these studies indicate that diverse genetic alterations converge on pathological mechanisms similar to those implicated in classical repeat diseases to cause ASD.

In addition to differences in repeat size and genetic variants in repair genes, we and others have shown that epigenetics is a predictor of age of disease onset and disease severity. Epigenetic marks such as methylation and chromatinization are increased at the repeat loci of patients with severe disease forms and early age of disease onset (Castaldo et al., 2008; Godler et al., 2010; Lanni et al., 2013; Xi et al., 2013; Naumann et al., 2014; De Souza et al., 2016; Barbe et al., 2017; Wang et al., 2017). Next, we discuss epigenetic marks and regulation at the repeat region of repeat diseases, and their influence on gene expression and disease phenotype.

## Methylation and CTCF Binding

DNA methylation is an epigenetic mark that changes the expression, regulation and organization of DNA. DNA methylation is essential for normal biological function and is associated with key processes important from early development to adulthood, such as X chromosome inactivation, gene imprinting, carcinogenesis and aging (Jones and Laird, 1999; Baylin et al., 2001; Robertson, 2001). Methylation occurs on cytosine residues, primarily those that are adjacent to guanine residues, and stretches of DNA with high incidence of CG sequences are most likely to be methylated. Such regions include promoters, enhancers and insulators, where methylation is typically associated with the inhibition of gene expression (Klose and Bird, 2006).

Stretches of hundreds of base pairs with a high percentage of CG sequences are called CpG islands. In many repeat disorders, the repeat expansions are located within or adjacent to CpG islands, thus DNA methylation can have an impact on disease phenotype. For many repeat diseases, CCCTC binding factor (CTCF) binding sites are located in the disease-associated CpG islands, and methylation results in an inhibition of CTCF binding. CTCF is a transcriptional repressor and often found at topologically associated domain (TAD) boundaries that regulate chromatin organization and gene expression (Nora et al., 2017). CTCF often co-localizes with cohesin, a protein important in the looping of the DNA during heterochromatin formation (Kagey et al., 2010).

Methylation up- and downstream of the CTG repeat has been noted in DM1 (Steinbach et al., 1998; Lopez Castel et al., 2011; Brouwer et al., 2013; Yanovsky-Dagan et al., 2015; Barbe et al., 2017), although almost exclusively in the most severe patients (Barbe et al., 2017). Two CTCF binding sites are located in the differentially methylated CpG islands up and downstream of the CTG repeat (Barbe et al., 2017; Franck et al., 2021), and are part of a regulatory element that controls *DMPK*, *DMWD*, and *SIX5* expression (Fu et al., 1993; Sabouri et al., 1993; Hamshere et al., 1997; Thornton et al., 1997; Alwazzan et al., 1999; Inukai et al., 2000; Narang et al., 2000; Depardon et al., 2001; Filippova et al., 2001; Yanovsky-Dagan et al., 2015). Methylation upstream of the CTG repeat region has exclusively been detected in blood, chorionic villi samples and human embryonic stem cells from individuals with maternal inheritance of the disease (Barbe et al., 2017). Maternal transmission is also biased toward longer CTG repeat sizes and more severe disease forms compared to paternal transmission (Harper et al., 1972; Harley et al., 1993; Lavedan et al., 1993; Pearson, 2003; Lagrue et al., 2019).

A methylation boundary upstream of the CGG repeat is lost in FXS (Naumann et al., 2009, 2014). This methylation boundary is located 65 CpG sites upstream of the repeat, at a CTCF binding site, and inactivates the promoter region when methylated, silencing *FMR1* (Oberle et al., 1991; Pieretti et al., 1991; Verkerk et al., 1991; Hansen et al., 1992; Sutcliffe et al., 1992; O’Donnell et al., 2002). Loss of FMR1 protein is the cause of FXS, and patients with atypically unmethylated promoter regions with full CGG repeat expansions are identified as “high functioning,” and lack FXS disease symptoms compared to patients with similar CGG expansions with methylation at the FMR1 promoter region, underscoring the importance of methylation in the disease manifestation (Godler et al., 2010; Lanni et al., 2013; Naumann et al., 2014). This understanding has led to the pursuit of methylation inhibitors, such as 5-aza-2-deoxycytidine, to reactivate FMR1 expression and potentially treat the disease (Chiurazzi et al., 1998; Pietrobono et al., 2002). However, toxicity has hampered the clinical roll-out of these approaches so far.

Methylation is increased in FRDA patients directly upstream of the GAA repeat and further upstream at a CpG island encompassing the promoter region, first exon and first intron of the *FXN* gene (Greene et al., 2007; Al-Mahdawi et al., 2008, 2013; Castaldo et al., 2008; vans-Galea et al., 2012; Quesada et al., 2015). CpG sites directly downstream of the repeat are hypomethylated in patients versus controls (Al-Mahdawi et al., 2008; vans-Galea et al., 2012). A subset of specific CpG sites in the *FXN* promoter and exon 1 are completely methylation-free in unaffected individuals but predominantly methylated in FRDA patients’ blood, brain, cerebellum and heart tissues (Greene et al., 2007; Al-Mahdawi et al., 2008). One of these CpG sites is located in a regulatory sequence that includes a CTCF binding sequence, and deletion of the regulatory sequence results in a drop in promoter activity (Greene et al., 2007), indicating that methylation could be responsible for gene expression changes. Increased levels of methylation are correlated with longer GAA repeats (Castaldo et al., 2008; vans-Galea et al., 2012) and earlier onset of disease (Castaldo et al., 2008).

Methylation is increased at the CpG island in the *C9ORF72* promoter in ALS patients with expanded C9ORF72 hexanucleotide G4C2 repeats (Xi et al., 2013; Russ et al., 2015; Gijselinck et al., 2016; Hamzeiy et al., 2018), and higher methylation levels are correlated with shorter disease duration (Xi et al., 2013) and longer repeat lengths (Gijselinck et al., 2016). Healthy individuals and patients with short repeat sizes do not show any methylation (Xi et al., 2013; Russ et al., 2015) in the C9ORF72 promoter. The CpG island downstream of the G4C2 repeat remains unmethylated in patients, similar to control individuals (Xi et al., 2013; Liu et al., 2014).

In HD, there is a CpG methylation boundary > 700 bp upstream of the *HTT* promoter, around 1200 bp upstream of the CAG repeat. The methylation boundary mostly stays intact in HD patients (Naumann et al., 2014). However, tissue-specific methylation differences between HD patients and controls have been found in the cortex, where differential methylation is found at a CTCF binding site in the *HTT* proximal promoter and increased DNA methylation is associated with earlier age of disease onset (De Souza et al., 2016; Lu et al., 2020).

The promoter regions of *ATXN2* and *ATXN3* are methylated in SCA2 and SCA3 patients with expanded repeats (Laffita-Mesa et al., 2012; Wang et al., 2017), and increased levels of methylation are found in the CTCF binding domains of *ATXN7* in SCA7 patients (Libby et al., 2008). In SCA3, methylation of the *ATXN3* promoter region is correlated with younger age of disease onset, and families with intergenerational CAG repeat instability exhibit higher methylation levels (Wang et al., 2017).

Whole-exome and whole-genome sequencing has revealed a higher incidence of mutations in genes that regulate DNA methylation in ASD patients compared to controls (Tremblay and Jiang, 2019). This results in multiple differentially methylated regions in autism, primarily at promoter CpG islands (Ladd-Acosta et al., 2014; Nardone et al., 2014). In ASD, differential methylation is also found at intragenic sites predicted to alter splicing, which results in changes to transcript isoforms (Maunakea et al., 2013; Irimia et al., 2014; Zhu et al., 2014; Quesnel-Vallieres et al., 2016).

In summary, classical repeat diseases are characterized by remarkably similar methylation patterns, with increased methylation upstream of the repeat region, often at CpG islands in or near the promoter region of the affected gene. Also, all of these differentially methylated regions harbor a CTCF binding site that is inhibited by methylation, which could in turn impact chromatinization and gene expression, as discussed below.

## Chromatin Organization and Gene Regulation

Chromatin organization regulates gene expression through DNA compaction. CTCF binding sites are disrupted upon increased methylation in repeat diseases, as discussed above, which results in chromatin changes in the repeat regions. In general, increased suppressing and decreased activating chromatin marks are found at the repeat regions for many repeat diseases, and disease-causing repeats are typically localized at TAD boundaries, which are disrupted by increased methylation and loss of CTCF binding. Chromatin compaction and loss of TAD boundaries results in gene downregulation in repeat diseases, which has been related to specific disease characteristics and symptoms.

In mouse models of DM1, a decrease in the active histone mark histone 3 lysine 9/14 acetylation (H3K9/14ac) and an enrichment of histone 3 lysine 27 trimethylation (H3K27me3) results in decreased expression of *DMPK* and *sine oculis* homeobox homolog 5 gene (*SIX5*) (Brouwer et al., 2013). In addition, an increase in H3K9 and H3K4 methylation is found in human DM1 fibroblast cell lines (Cho et al., 2005). This altered chromatin pattern toward compaction was confirmed in disease-relevant tissues such as myoblasts, myotubes, skeletal muscle, heart, lung and osteoblasts (Buckley et al., 2016), and accordingly expression of *DMPK* and *SIX5* has been reported to be decreased in these tissues in DM1 patients (Fu et al., 1993; Hofmann-Radvanyi and Junien, 1993; Sabouri et al., 1993; Thornton et al., 1997; Alwazzan et al., 1999; Eriksson et al., 2000, 2001; Buckley et al., 2016). DMPK is involved in muscle function, and decreased *DMPK* expression has been linked to muscle impairment and cardiac disease in patients and mouse models (Benders et al., 1997; Berul et al., 1999; Mounsey et al., 2000). A decrease of *SIX5* expression is thought to cause cataracts in DM1 patients (Thornton et al., 1997; Alwazzan et al., 1999; Klesert et al., 2000; Sarkar et al., 2000). Male infertility has been associated with two genes further upstream in the repeat region, *DMWD* and *RSPH6A*, for which transcription is also reduced in DM1 patients (Shaw et al., 1993; Jansen et al., 1996; Alwazzan et al., 1999; Eriksson et al., 2001). Finally, a TAD boundary at the repeat region and CTCF binding site was recently shown to be disrupted in DM1, indicating that chromatin organization may be more broadly altered in the disease (Sun et al., 2018).

In FXS, expansion of the CGG repeat to > 200 repeats leads to a chromatin reorganization from euchromatin to heterochromatin. In this setting, heterochromatin marks H3K27me3, H4K20me3 and H3K9me2/3 are increased, and euchromatin marks H3 and H4 acetylation and H3K4me2 are decreased (Coffee et al., 1999, 2002; Gheldof et al., 2006; Kumari and Usdin, 2010). These chromatin changes result in the loss of a TAD boundary near the *FMR1* gene, leading to dysregulated 3D chromatin structure with decreased genomic interactions directly at and downstream of the repeat region, and increased interactions of the *FMR1* gene with upstream gene regions (Gheldof et al., 2006; Sun et al., 2018). The loss of the TAD boundary has directly been linked with the loss of CTCF binding at the *FMR1* promoter region (Sun et al., 2018). As a result, *FMR1* is silenced, and indeed reduced FMR1 protein is found in patient lymphoblast cells (Coffee et al., 1999). In a cell model, treatment with 5-aza-2′-deoxycytidine reinitiated acetylation on histones H3 and H4 and transcriptional reactivation of the *FMR1* gene in lymphoblastoid cell lines (Coffee et al., 1999; Tabolacci et al., 2005; Biacsi et al., 2008). Interestingly, RNA-DNA duplexes of the expanded RNA binding to the repeat expansion in the DNA have been linked to silencing of the *FMR1* gene, and blockage of this interaction reverts chromatin marks to a euchromatin state and reactivates *FMR1* expression (Colak et al., 2014).

The GAA repeat region in FRDA is located near a TAD boundary, which is lost upon repeat expansion in FRDA patients (Sun et al., 2018). This is accompanied by loss of activating chromatin marks including acetylation of histones H3 and H4 (Herman et al., 2006). Heterochromatin marks H3K9me2/3 and H3K27me3 are increased in the repeat region, and the *FXN* gene is downregulated in patient cell lines, brain tissue and mouse models of FRDA (Greene et al., 2007; Al-Mahdawi et al., 2008; De Biase et al., 2009). Treatment of FRDA patient lymphoblast cells and FRDA mouse models with deacetylase inhibitors increases *FXN* expression (Herman et al., 2006; Rai et al., 2008; Sandi et al., 2011), suggesting a primary role of chromatin compaction in the regulation of FXN gene expression in disease. Also, removal of the full intron 1 sequence rather than the repeat region only was able to restore cellular and molecular deficits of the disease in dorsal root ganglia organoids, again suggesting that chromatin compaction of the larger region is involved in disease pathogenesis (Mazzara et al., 2020).

In *C9ORF72* familial cases of ALS, there is also an increase in chromatin suppressive marks such as trimethylation at residues H3K9, H3K27, H3K79 and H4K20 (Belzil et al., 2013) and a TAD boundary is located at the *C9ORF72* locus (Sun et al., 2018). *C9ORF72* transcript and protein levels are downregulated in frontal cortices and cerebelli of *C9ORF72* familial cases of ALS patients (DeJesus-Hernandez et al., 2011; Gijselinck et al., 2012; Belzil et al., 2013; Waite et al., 2014) and methylation inhibition of the chromatin lysine residues decreases methylation at the repeat region and restores *C9ORF72* transcript levels in patient fibroblast cell lines (DeJesus-Hernandez et al., 2011; Gijselinck et al., 2012; Belzil et al., 2013).

In HD, a TAD boundary is located at the differentially methylated CTCF site upstream of the repeat which remodels chromatin compaction and can change gene-enhancer interactions (Sun et al., 2018). Although overall *HTT* levels are typically not reported to be downregulated in HD patients, supplementation of wild type *HTT* does decrease neuronal toxicity in mice (Leavitt et al., 2001; Thomson and Leavitt, 2018). Therefore, finding a balance between mutant and wild type *HTT* levels is key, and mutant *HTT* lowering strategies are well underway as an HD therapeutic.

A disrupted TAD boundary was recently identified at the repeat region of the *ATXN1* gene in SCA1 patients (Sun et al., 2018) but an understanding of the chromatin regulation of the other spinocerebellar ataxia-causing genes remains lacking.

While ASD can have different genetic causes, the level of heterochromatin mark H3K4me3 (Shulha et al., 2012) and acetylation of H3K27 (Sun et al., 2016) are overall increased in post-mortem brain samples from patients with ASD.

**FIGURE 1:**
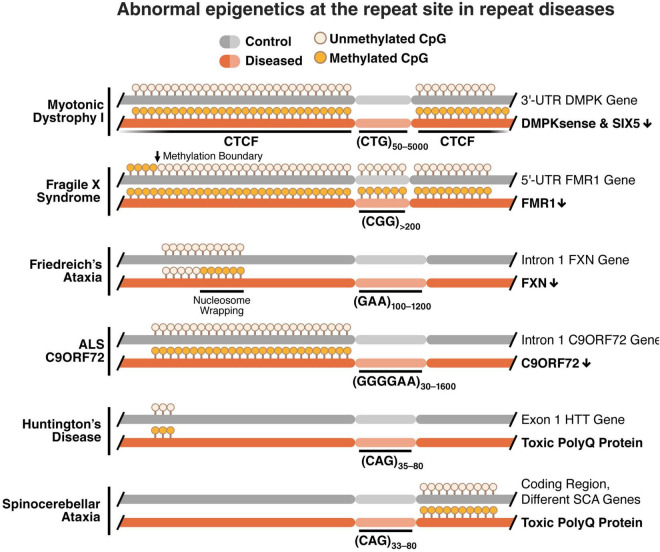
Methylation of the repeat locus is associated with epigenetic deregulation of the nucleotide repeat region with a loss in CTCF binding, loss of TAD boundaries, chromatin condensation, and gene down regulation. Increased methylation is typically found near the repeat region in CG-rich areas called CpG islands. These differentially methylated regions include CTCF binding sites, and methylation inhibits binding of the CTCF protein. With a loss of CTCF binding, TAD boundaries are also lost and chromatin folding is altered to a more condensed formation. This leads to down regulation of genes with promoters and enhancers at or near the repeat region in several repeat diseases.

Taken together, these studies indicate a common epigenetic signature across repeat diseases characterized by increased DNA methylation, loss of CTCF binding, loss of TAD boundaries and increased chromatinization at the repeat region (Figure 1).

Different epigenetic states may in part underlie the variability in disease manifestation across individuals with the same disease-causing mutation, and since environmental input can alter epigenetic state, it can also affect disease severity. There are several examples of such associations in the literature, such as the finding that cage enrichment delays disease onset in mouse models of HD (Van Dellen et al., 2000). Similar results were found in a mouse model of FXS, in which mice housed with cage enrichment showed behavioral and neuronal morphological recovery (Restivo et al., 2005). However, how these environmental exposures trigger epigenetic changes that interact with genetic mutations and affect disease phenotype are largely unknown.

The common signature of epigenetic changes that we found in classical repeat diseases are associated with disease severity and age of disease onset. Down regulation of genes in the repeat region could be one disease mechanism through which the epigenetic signature changes disease phenotype. Additionally, the epigenetic signature at the repeat region could directly or indirectly interact with the DNA and cause disease phenotypes through repeat instability, as discussed next.

## Genetic and Epigenetic Interplay Through Repeat Instability

Repeat expansions are a critical element of repeat diseases. However, the repeat size alone does not explain the variation in disease severity and age of disease onset (Cobo et al., 1993; Barcelo et al., 1994; Clark et al., 1998; Langbehn et al., 2004; Wexler et al., 2004; De Antonio et al., 2016; Keum et al., 2016), and other factors like epigenetics may be better predictors of disease phenotype (Barbe et al., 2017). However, how genetics and epigenetics might interact to cause disease remains unknown.

**FIGURE 2:**
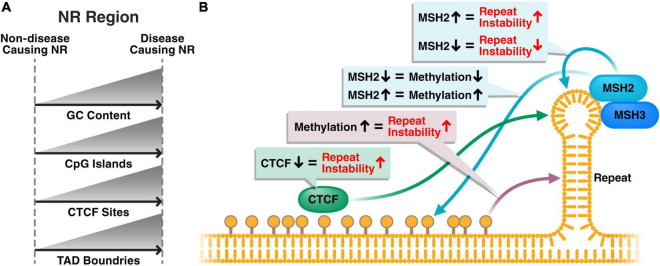
Hypothetical disease model of the role of the common epigenetic signature in repeat instability and disease phenotype. **(A)** Disease-causing nucleotide repeats typically have higher GC content, more CpG islands and more CTCF sites compared to non-disease-causing repeats in the genome. Typically, a TAD boundary will be present near disease-causing repeats. **(B)** Methylation near the nucleotide repeat results in increased repeat instability, potentially through a loss of CTCF binding, which is also associated with increased repeat instability. Additionally, the mismatch repair pathway, of which MSH2 and MSH3 are the main players at repeats, interacts with the hairpin structures formed at the repeat region and causes repeat instability. The close proximity of methylation and mismatch repair proteins to repeat regions could suggest that epigenetics and the mismatch repair machinery play a cooperative role regulating repeat instability.

Repeat instability, whereby the repeat size expands and contracts within cells over time, has been identified in most repeat diseases. Overall, repeat instability and repeat expansions are higher in affected tissues and cell types (Anvret et al., 1993; Ashizawa et al., 1993; Thornton et al., 1994; Chong et al., 1995; Dobkin et al., 1996; Takano et al., 1996; Watanabe et al., 2000; Kennedy et al., 2003; De Biase et al., 2007; Nordin et al., 2015; Mouro Pinto et al., 2020). Repeat regions show an increase in secondary DNA structures, with increased DNA folding into DNA loops (Schmidt and Pearson, 2016). Repeat instability is thought to be caused by the incorrect repair of these secondary structures by mismatch repair proteins (Schmidt and Pearson, 2016). Whether the common epigenetic signature we describe in this review also plays a role in the formation of these secondary structures and repeat instability remains largely unknown (Figure 2).

Brock et al. (1999) identified repeat diseases to originate from loci rich in GC content. They found a strong correlation between “expandability” of the repeat and the GC content in the flanking regions, and showed that the most expandable repeats were located near CpG islands. Similarly, although repeat expansions happen throughout the genome, repeat diseases are primarily caused by expansions near a TAD domain, often located in GC-rich DNA sequences with CTCF binding sites (Sun et al., 2018).

In addition to altering expression of disease-causing genes, changes in DNA methylation may also enhance neurodegeneration and other neuronal disease phenotypes in repeat disease by increasing repeat instability and expansion. For example, knockdown of DNA methyltransferase 1 (DNMT1) in human cells and mouse models of SCA1 results in an increase in repeat instability (Dion et al., 2008). Additionally, high levels of DNA methylation upstream of the CGG repeat in the ovaries of SCA1 mice were correlated with low frequencies of stable repeat transmission to offspring (Dion et al., 2008). Franck et al. (2021) recently reported that downregulation of mismatch repair proteins, known to bind DNA loops formed at expanded CTG repeats, are associated with repeat stabilization and a loss of methylation at the repeat region in DM1 (Franck et al., 2021). They found that re-introduction of the mismatch repair proteins re-initiated repeat instability and partially restored methylation (Franck et al., 2021). These results provide direct evidence of an interplay between methylation and repeat instability at the expanded repeat region, however, a causal link between methylation and repeat instability has not been assessed.

CTCF binding is inhibited upon methylation of the CTCF binding site, which is thought to have an effect on chromatinization and thereby gene expression. Additionally, repeat instability has been linked to a loss of CTCF binding as a consequence of mutations in CTCF binding sites in SCA7 transgenic mouse models (Libby et al., 2008).

Many questions remain regarding the involvement of epigenetics in gene regulation, repeat instability and the consequences for cognitive symptoms and neurodegeneration. To address these questions, models that retain relevant epigenetic marks in disease-relevant cell types are necessary. Next, we discuss the disease models that are currently available to study the role of epigenetics in disease phenotype and how these compare to human pluripotent stem cell models.

## Epigenetic Disease Modeling for Neurological Dysfunction in Repeat Diseases

Most current models for repeat diseases, including human induced pluripotent stem cell (hiPSC)-derived cells and animal models, primarily model the genetic aspects of the disease.

Animals are useful to model distinct sets of repeat disease pathologies and phenotypes. Because most repeat diseases do not naturally occur in rodents, animal models of the diseases are generally made by knocking-in abnormally large repeat lengths, and the resulting animals generally show fewer symptoms than typical patients (Jansen et al., 1996; Mangiarini et al., 1996; Matilla et al., 1998; Klesert et al., 2000; Sarkar et al., 2000; Puccio et al., 2001; Watase et al., 2002, 2008; Simon et al., 2004; Gomes-Pereira et al., 2007; Panaite et al., 2008; Menalled et al., 2009; Kazdoba et al., 2014; Chew et al., 2015; Jiang et al., 2016; Liu Y. et al., 2016). Alternatively, knock down or knock out models are used to eliminate expression of the disease-causing genes in DM1, FMR1 and SCA1 (Fu et al., 1993; The Dutch-Belgian Fragile X Consorthium et al., 1994; Matilla et al., 1998; Klesert et al., 2000; Huguet et al., 2012; Kazdoba et al., 2014). While these models have been useful to understand disease pathways and phenotypes, the epigenetic marks associated with the human diseases are often not expressed because of the knock-in and knock-down techniques used to induce repeat diseases in mice (Kovtun and McMurray, 2008). Therefore, these models are unfit to study the role epigenetic changes near the repeat region might have on disease.

Human post-mortem samples are widely used to study phenotypes related to repeat length (Greco et al., 2011; Lopez Castel et al., 2011; Seidel et al., 2012; Waldvogel et al., 2012; Esanov et al., 2016). However, these samples are typically only available from the most severe, fatal forms of the diseases, and give little information about early disease mechanisms that could be targeted with drugs. As an alternative, many research groups investigate repeat diseases using hiPSCs generated from fibroblasts or blood samples of patients. This extends the breadth of disease severities that can be studied, as well as the possibility to study family members who do not express disease phenotypes. Also, gene-corrected cell lines can be generated from the patient cell lines to specifically determine the effect of the repeat expansion in the same genetic background. However, modeling epigenetics using hiPSC models has been challenging because of the potential absence or loss of epigenetic and aging signatures during cell reprogramming to a pluripotent state (Tang et al., 2017; Victor et al., 2018).

Challenges to studying epigenetics in repeat diseases, and aging diseases overall, remain due to the inability of animal models to recapitulate disease phenotypes at human repeat lengths, the absence of temporal information and early disease phenotypes in post-mortem samples and the absence of epigenetic and aging signatures in hiPSC models (Patterson et al., 2012). Thus, models that retain epigenetic and aging marks have been proposed as alternative approaches to study the role of epigenetics and aging in repeat diseases and other neurodegenerative diseases.

Direct conversion of adult cells, also called transdifferentiation, is typically done from fibroblasts generated from skin biopsies (Vierbuchen et al., 2010) or from blood cells (Tanabe et al., 2018), and the method is furthest developed for neuronal conversion (Vierbuchen et al., 2010). Adult cells are converted to neurons with the use of small molecules, neuronal transcription factors, micro-RNAs and epigenetic modifiers and bypass the pluripotent stage by going through a unique intermediate state without transcriptional specification of either the donor or the target cell type (Treutlein et al., 2016; Chen et al., 2019). Converted neurons retain many of the epigenetic marks from the fibroblast state (Huh et al., 2016), and also appear to generate new epigenetic marks typical for the neuronal lineage (Luo et al., 2019; Traxler et al., 2019). The converted neurons have the same molecular age as the fibroblasts they originated from, as determined by DNA methylation marks of the epigenetic clock (Horvath, 2013; Huh et al., 2016). Converted neurons also retain other cellular properties of aging such as shorter telomere lengths, chromatin and nuclear organization, higher levels of DNA damage and increased oxidative stress (Huh et al., 2016; Tang et al., 2017), whereas hiPSC-derived neurons, generated from the same fibroblasts, adopt a younger, more embryonic-like state (Tang et al., 2017).

One approach to investigate epigenetic signatures in human cell models is to use direct conversion of patient cells to disease-relevant cell types. Direct conversion substantially retains the epigenetic signatures of the adult cell, an advantage for modeling diseases related to epigenetics and aging (Traxler et al., 2019). The direct conversion approach could also enable disease modeling at repeat sizes known to cause disease, with the flexibility to use samples from patients with different disease severities, and potentially without erasing epigenetic disease marks at the repeat region to enable studies of drug interventions and cause-effect relationships in cell culture. Epigenetic marks are generally established and maintained by enzymes and thus these enzymes are potentially druggable. For example, there are already FDA-approved cancer drugs that target epigenetic states including DNA methylation, chromatin remodeling and non-coding RNAs (Ghasemi, 2020). Additionally, these human cell models created by transdifferentiation could be used to study the impact of aging on disease phenotypes in which aging is a risk factor, such as HD and ALS.

A few studies have modeled repeat diseases with directly converted neurons and demonstrated disease phenotypes (Jovicic et al., 2015; Liu M. L. et al., 2016; Victor et al., 2018). For example, Jovicic et al. (2015) found that directly converted neurons from ALS patients showed mislocalization of the regulator of chromosome condensation 1 (RCC1) protein, a Ran guanine nucleotide exchange factor they used to determine nucleocytoplasmic exchange. Additionally, Liu et al. (Liu M. L. et al., 2016) found increased neurodegeneration of directly converted motor neurons from ALS patients, as evidenced by poor cell survival, a loss of neuromuscular junctions, reduced cell activity and soma shrinkage.

One study nicely compared HD phenotypes found in medium spiny neurons (MSNs) directly converted from fibroblasts to phenotypes in MSNs derived from iPSC-derived fibroblasts (Victor et al., 2018). This study showed that the directly converted MSNs expressed disease phenotypes that have been difficult to demonstrate in hiPSC-derived MSNs, including Huntingtin aggregates, a disease biomarker found in patient samples. In addition, the authors showed that cellular disease phenotypes were enhanced in the directly converted MSNs, such as increased DNA damage levels, mitochondrial dysfunction and neurodegeneration (Victor et al., 2018).

These recent findings suggest that modeling repeat diseases with directly converted neurons may be preferred over hiPSC-derived neuronal cell models because of the increased number of disease phenotypes that can be detected, and because directly converted neurons express disease phenotypes that are similar to those observed in post-mortem patient samples. Moreover, a common epigenetic signature is present across repeat diseases, and disease models that retain epigenetic signatures are necessary to investigate the underlying mechanisms.

## Conclusion

Repeat diseases are caused by genetic repeat expansions. However, the variation in age of disease onset and severity in repeat disease patients cannot be explained solely by differences in repeat size. Here, we describe a common epigenetic signature across repeat diseases, found to be correlated with disease severity and age of onset. This signature includes DNA methylation near the repeat region, including at CTCF binding sites, which results in the loss of CTCF binding, loss of a TAD boundary and increased chromatinization. This leads to altered expression of genes in the repeat region which can cause specific disease symptoms. Additionally, an interplay between genetics and epigenetics at the repeat region suggests that methylation contributes to repeat instability. Notably, these mechanisms likely also contribute to some polygenic disorders, such as ASD. Unfortunately, a broad understanding of how an altered epigenetic state can cause cellular dysfunction remains lacking, largely due to the inability to study cause-and-effect relationships in most current disease models. However, direct conversion of fibroblasts to disease-relevant cell types, with the retention of patient epigenetic and aging signatures, has set the stage to unravel the mechanisms by which epigenetic changes can cause disease phenotypes and study drug interventions in repeat diseases.

## Author Contributions

LB and SF: conceptualization, writing–review, editing, and funding acquisition. LB: writing–original draft. Both authors contributed to the article and approved the submitted version.

## Conflict of Interest

The authors declare that the research was conducted in the absence of any commercial or financial relationships that could be construed as a potential conflict of interest.

## Publisher’s Note

All claims expressed in this article are solely those of the authors and do not necessarily represent those of their affiliated organizations, or those of the publisher, the editors and the reviewers. Any product that may be evaluated in this article, or claim that may be made by its manufacturer, is not guaranteed or endorsed by the publisher.
